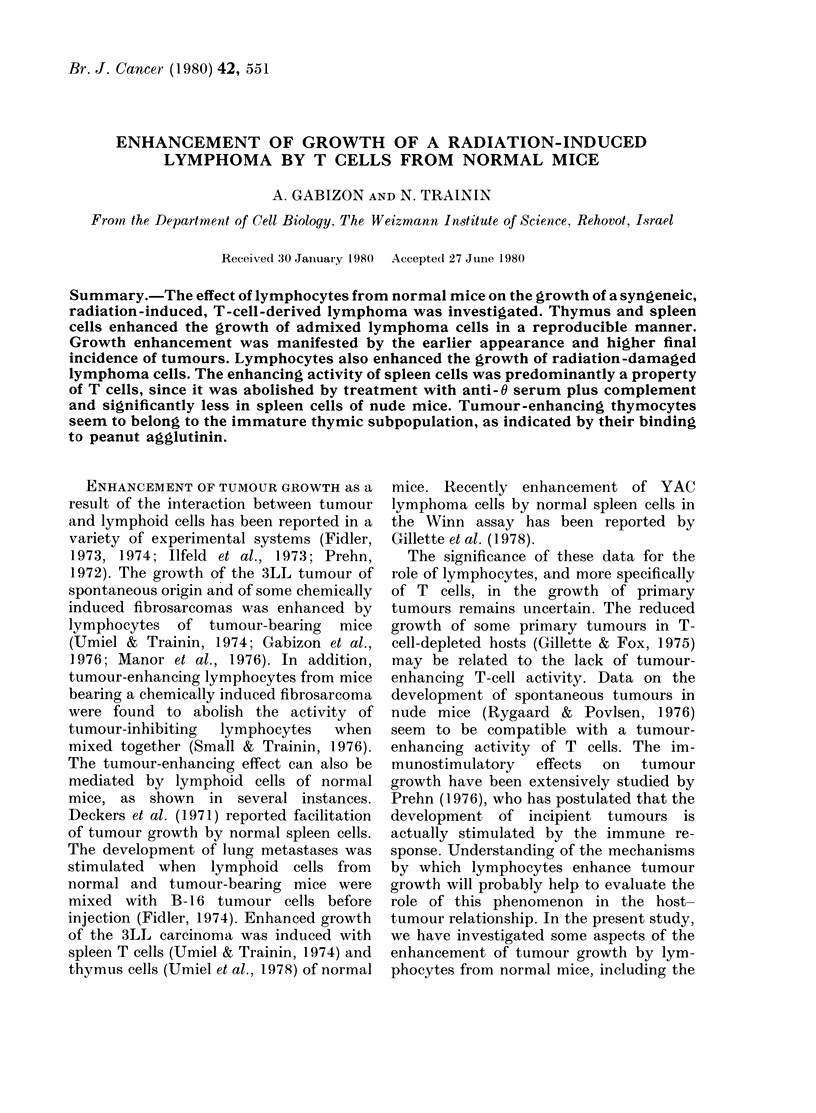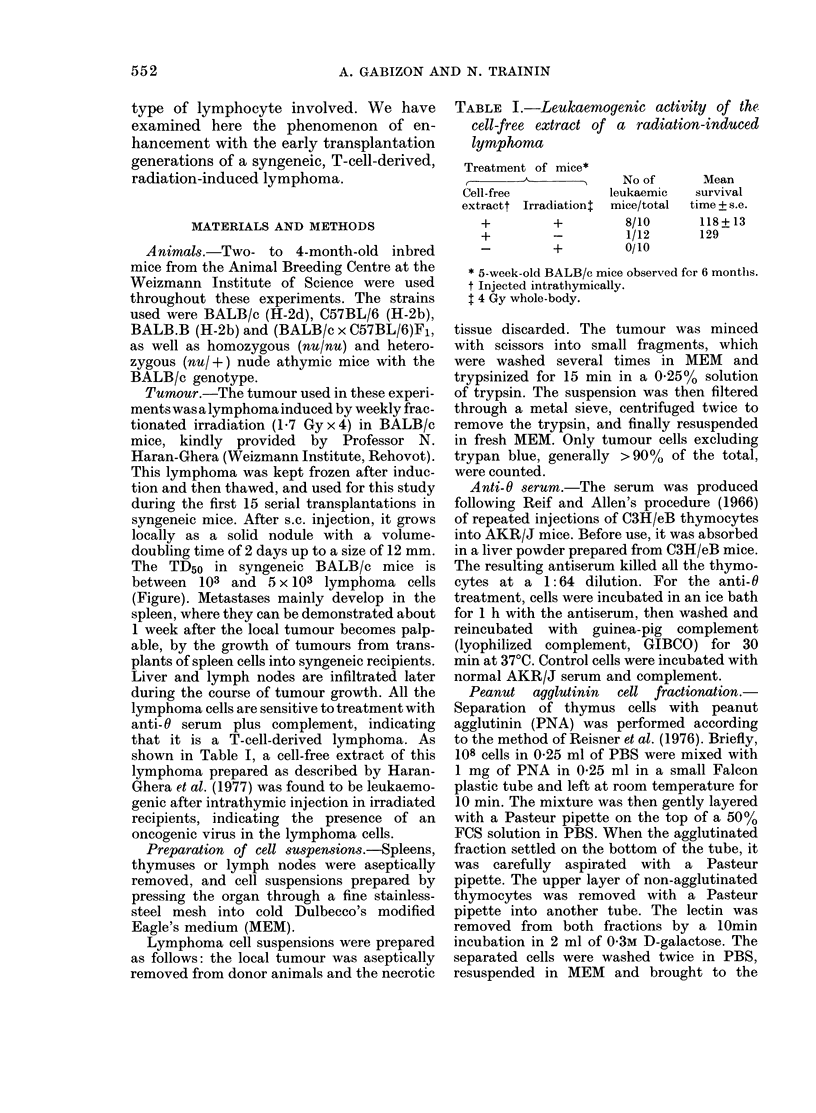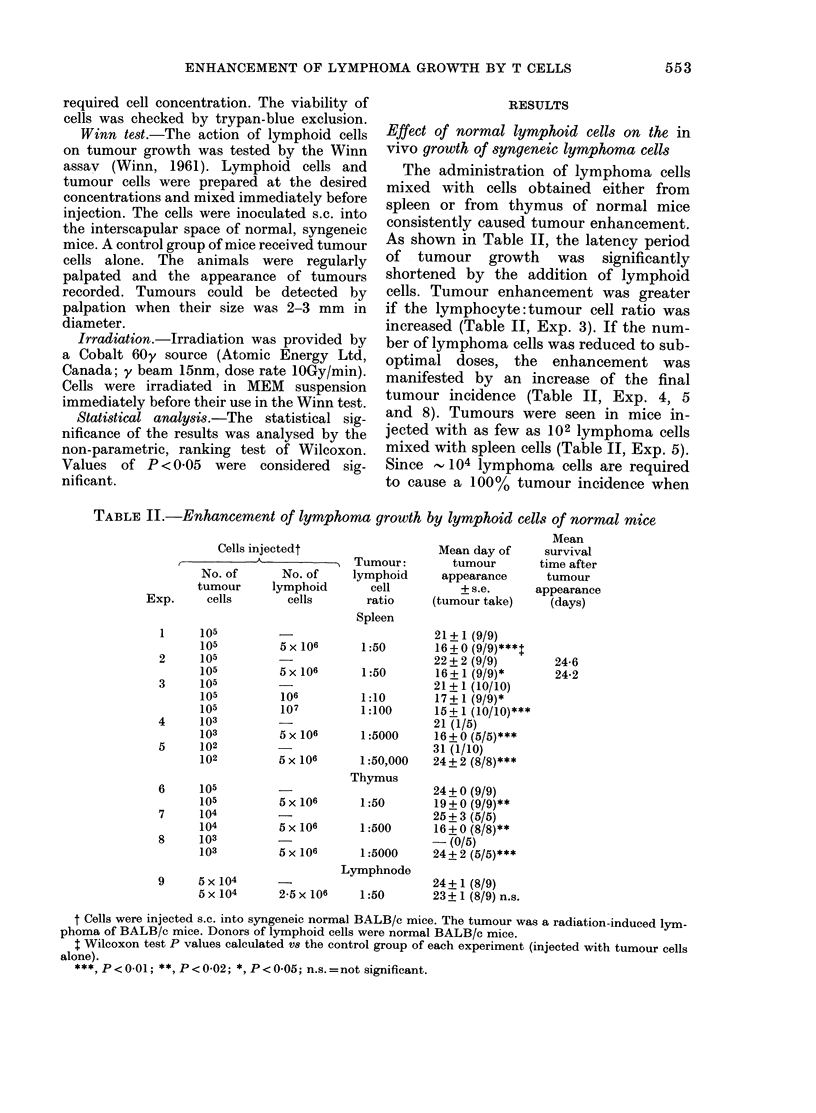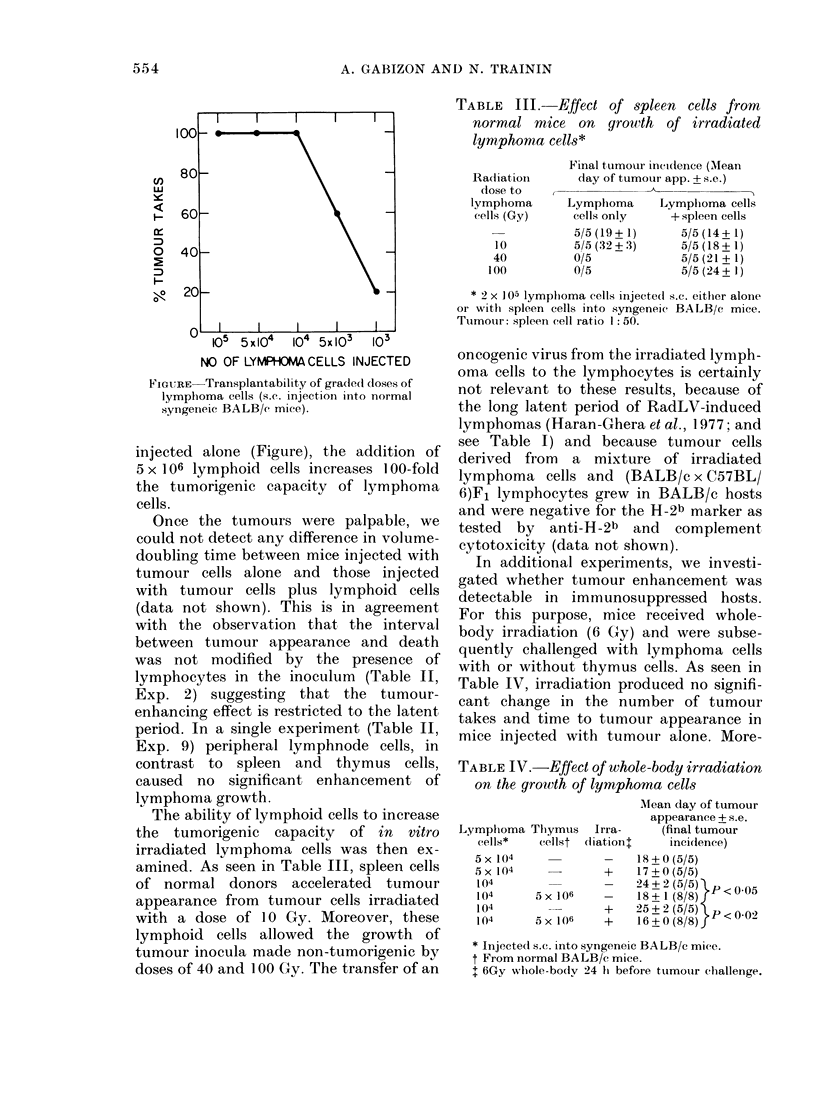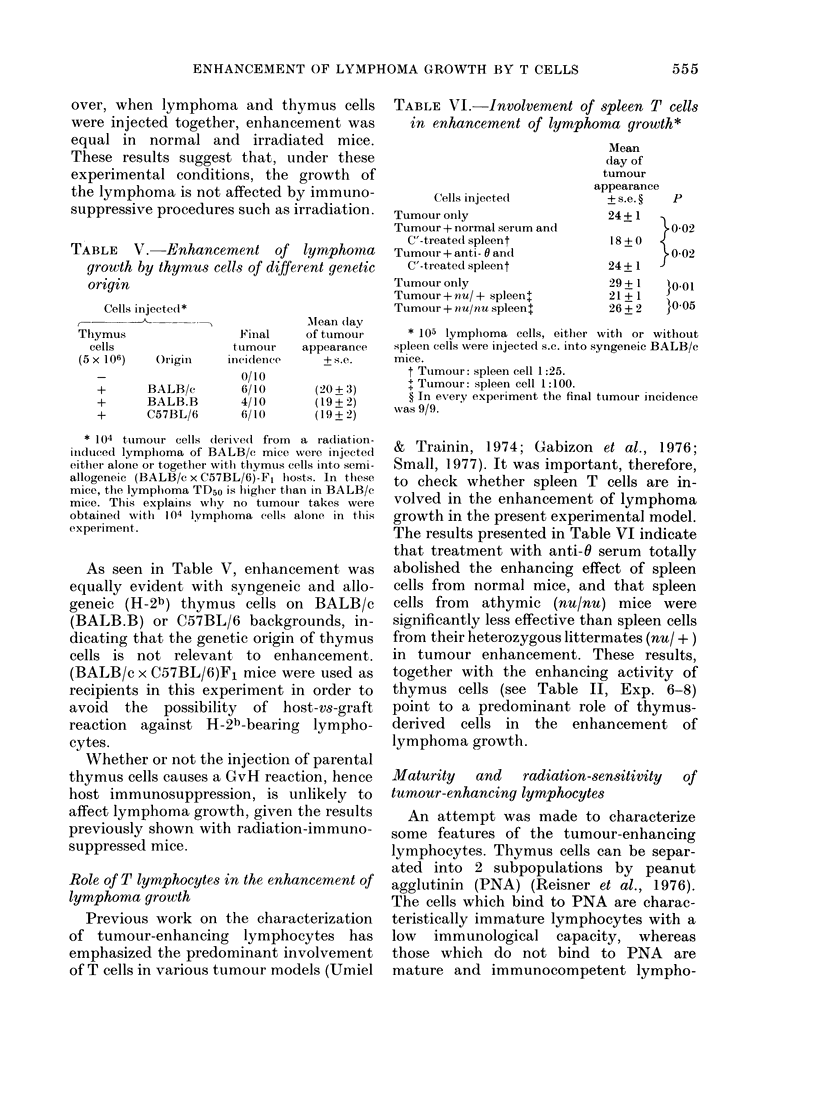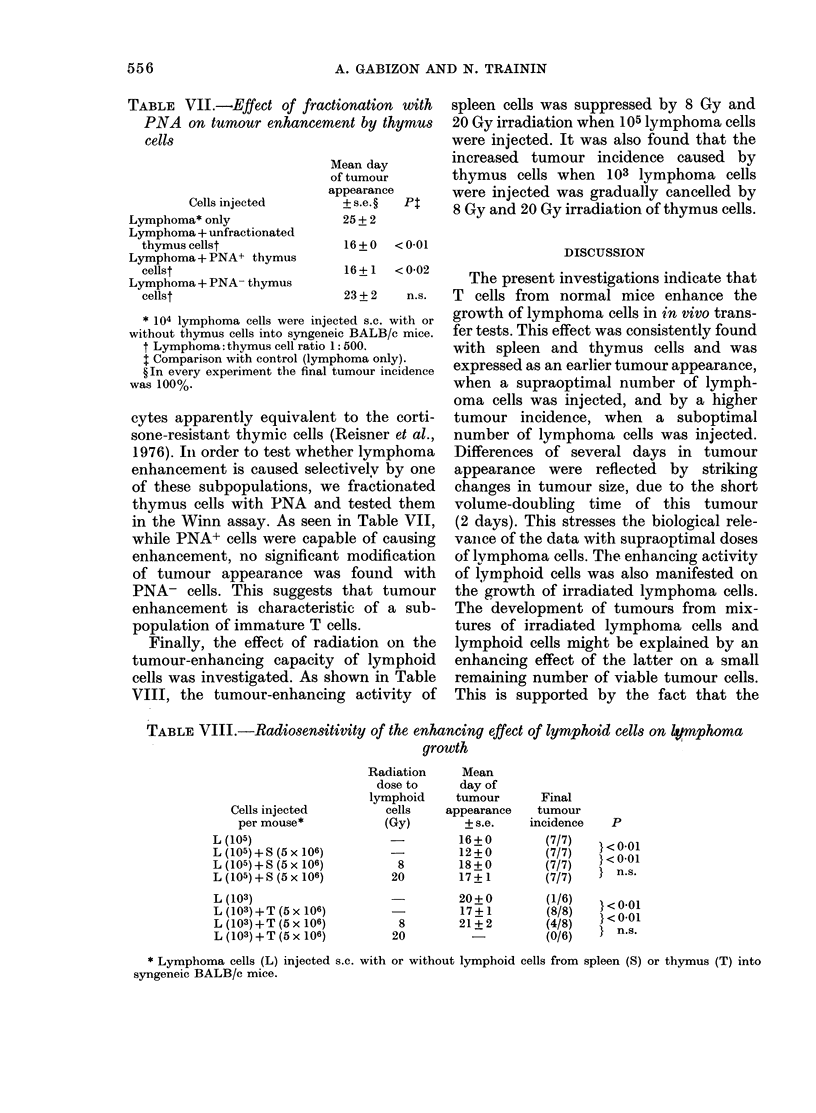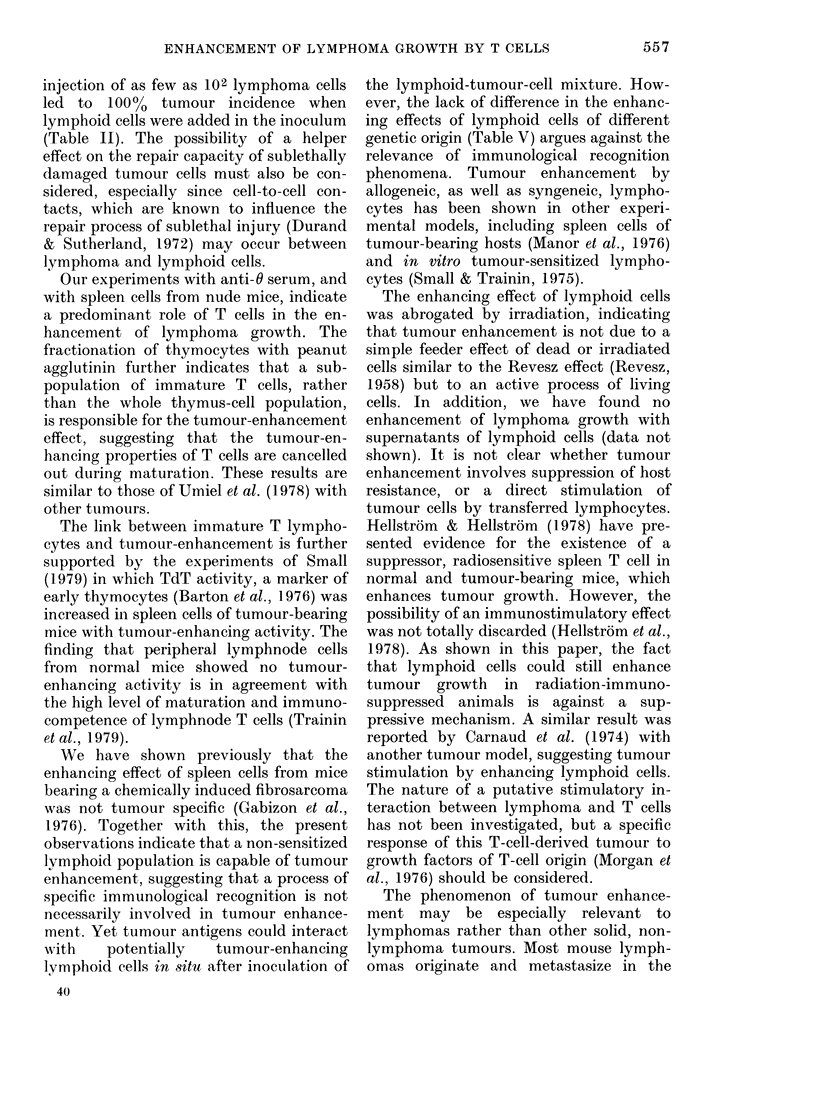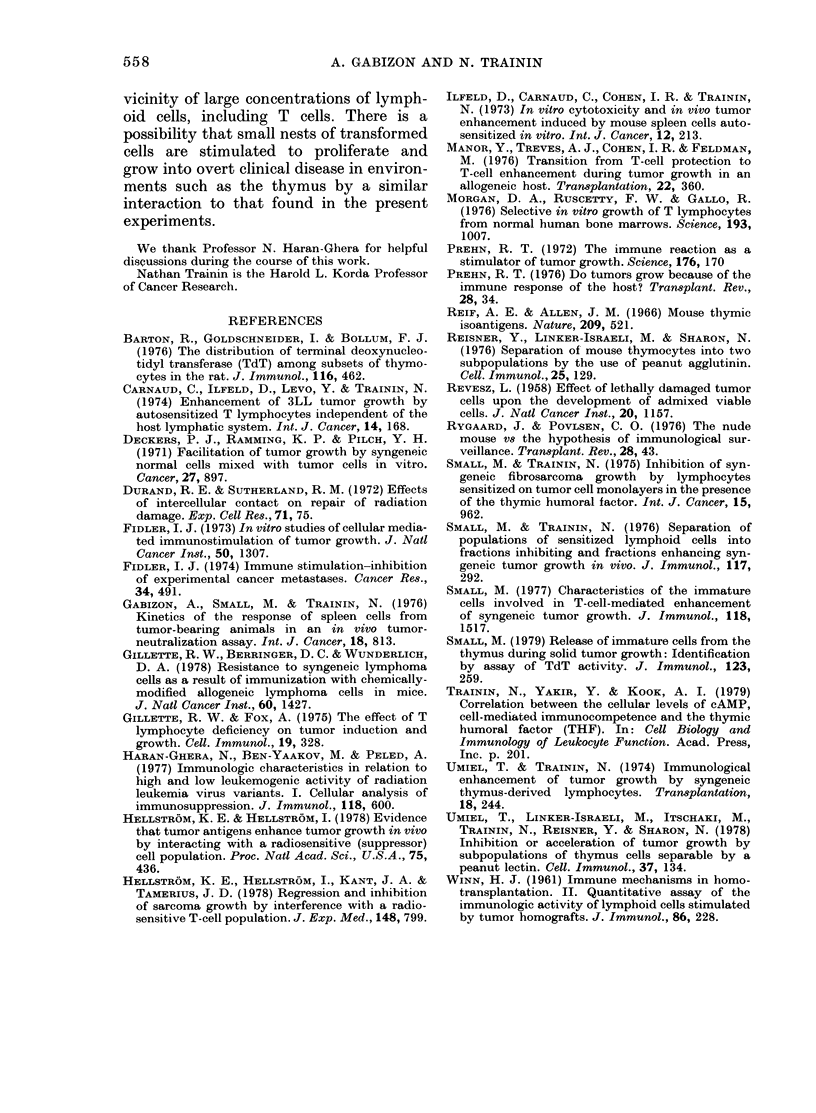# Enhancement of growth of a radiation-induced lymphoma by T cells from normal mice.

**DOI:** 10.1038/bjc.1980.279

**Published:** 1980-10

**Authors:** A. Gabizon, N. Trainin

## Abstract

The effect of lymphocytes from normal mice on the growth of a syngeneic, radiation-induced, T-cell-derived lymphoma was investigated. Thymus and spleen cells enhanced the growth of admixed lymphoma cells in a reproducible manner. Growth enhancement was manifested by the earlier appearance and higher final incidence of tumours. Lymphocytes also enhanced the growth of radiation-damaged lymphoma cells. The enhancing activity of spleen cells was predominantly a property of T cells, since it was abolished by treatment with anti-theta serum plus complement and significantly less in spleen cells of nude mice. Tumour-enhancing thymocytes seem to belong to the immature thymic subpopulation, as indicated by their binding to peanut agglutinin.


*Br. J. Cancer* (1980) **42**, 551

### ENHANCEMENT OF GROWTH OF A RADIATION-INDUCED LYMPHOMA BY T CELLS FROM NORMAL MICE

A. GABIZON AND N. TRAININ

*From the Department of Cell Biology, The Weizmann Institute of Science, Rehovot, Israel*

Received 30 January 1980 Accepted 27 June 1980

**Summary.—The effect of lymphocytes from normal mice on the growth of a syngeneic, radiation-induced, T-cell-derived lymphoma was investigated. Thymus and spleen cells enhanced the growth of admixed lymphoma cells in a reproducible manner. Growth enhancement was manifested by the earlier appearance and higher final incidence of tumours. Lymphocytes also enhanced the growth of radiation-damaged lymphoma cells. The enhancing activity of spleen cells was predominantly a property of T cells, since it was abolished by treatment with anti-$\theta$ serum plus complement and significantly less in spleen cells of nude mice. Tumour-enhancing thymocytes seem to belong to the immature thymic subpopulation, as indicated by their binding to peanut agglutinin.**

ENHANCEMENT OF TUMOUR GROWTH as a result of the interaction between tumour and lymphoid cells has been reported in a variety of experimental systems (Fidler, 1973, 1974; Ilfeld *et al.*, 1973; Prehn, 1972). The growth of the 3LL tumour of spontaneous origin and of some chemically induced fibrosarcomas was enhanced by lymphocytes of tumour-bearing mice (Umiel & Trainin, 1974; Gabizon *et al.*, 1976; Manor *et al.*, 1976). In addition, tumour-enhancing lymphocytes from mice bearing a chemically induced fibrosarcoma were found to abolish the activity of tumour-inhibiting lymphocytes when mixed together (Small & Trainin, 1976). The tumour-enhancing effect can also be mediated by lymphoid cells of normal mice, as shown in several instances. Deckers *et al.* (1971) reported facilitation of tumour growth by normal spleen cells. The development of lung metastases was stimulated when lymphoid cells from normal and tumour-bearing mice were mixed with B-16 tumour cells before injection (Fidler, 1974). Enhanced growth of the 3LL carcinoma was induced with spleen T cells (Umiel & Trainin, 1974) and thymus cells (Umiel *et al.*, 1978) of normal mice. Recently enhancement of YAC lymphoma cells by normal spleen cells in the Winn assay has been reported by Gillette *et al.* (1978).

The significance of these data for the role of lymphocytes, and more specifically of T cells, in the growth of primary tumours remains uncertain. The reduced growth of some primary tumours in T-cell-depleted hosts (Gillette & Fox, 1975) may be related to the lack of tumour-enhancing T-cell activity. Data on the development of spontaneous tumours in nude mice (Rygaard & Povlsen, 1976) seem to be compatible with a tumour-enhancing activity of T cells. The immunostimulatory effects on tumour growth have been extensively studied by Prehn (1976), who has postulated that the development of incipient tumours is actually stimulated by the immune response. Understanding of the mechanisms by which lymphocytes enhance tumour growth will probably help to evaluate the role of this phenomenon in the host–tumour relationship. In the present study, we have investigated some aspects of the enhancement of tumour growth by lymphocytes from normal mice, including the type of lymphocyte involved. We have examined here the phenomenon of enhancement with the early transplantation generations of a syngeneic, T-cell-derived, radiation-induced lymphoma.

### MATERIALS AND METHODS

*Animals.*—Two- to 4-month-old inbred mice from the Animal Breeding Centre at the Weizmann Institute of Science were used throughout these experiments. The strains used were BALB/c (H-2d), C57BL/6 (H-2b), BALB.B (H-2b) and (BALB/c × C57BL/6)$F_1$, as well as homozygous (*nu/nu*) and heterozygous (*nu*/+) nude athymic mice with the BALB/c genotype.

*Tumour.*—The tumour used in these experiments was a lymphoma induced by weekly fractionated irradiation (1·7 Gy × 4) in BALB/c mice, kindly provided by Professor N. Haran-Ghera (Weizmann Institute, Rehovot). This lymphoma was kept frozen after induction and then thawed, and used for this study during the first 15 serial transplantations in syngeneic mice. After s.c. injection, it grows locally as a solid nodule with a volume-doubling time of 2 days up to a size of 12 mm. The $TD_{50}$ in syngeneic BALB/c mice is between $10^3$ and $5 \times 10^3$ lymphoma cells (Figure). Metastases mainly develop in the spleen, where they can be demonstrated about 1 week after the local tumour becomes palpable, by the growth of tumours from transplants of spleen cells into syngeneic recipients. Liver and lymph nodes are infiltrated later during the course of tumour growth. All the lymphoma cells are sensitive to treatment with anti-$\theta$ serum plus complement, indicating that it is a T-cell-derived lymphoma. As shown in Table I, a cell-free extract of this lymphoma prepared as described by Haran-Ghera *et al.* (1977) was found to be leukaemogenic after intrathymic injection in irradiated recipients, indicating the presence of an oncogenic virus in the lymphoma cells.

*Preparation of cell suspensions.*—Spleens, thymuses or lymph nodes were aseptically removed, and cell suspensions prepared by pressing the organ through a fine stainless-steel mesh into cold Dulbecco's modified Eagle's medium (MEM).

Lymphoma cell suspensions were prepared as follows: the local tumour was aseptically removed from donor animals and the necrotic tissue discarded. The tumour was minced with scissors into small fragments, which were washed several times in MEM and trypsinized for 15 min in a 0·25% solution of trypsin. The suspension was then filtered through a metal sieve, centrifuged twice to remove the trypsin, and finally resuspended in fresh MEM. Only tumour cells excluding trypan blue, generally >90% of the total, were counted.

TABLE I.—*Leukaemogenic activity of the cell-free extract of a radiation-induced lymphoma*

| Treatment of mice* | | | |
|---|---|---|---|
| Cell-free extract† | Irradiation‡ | No of leukaemic mice/total | Mean survival time ± s.e. |
| + | + | 8/10 | 118 ± 13 |
| + | − | 1/12 | 129 |
| − | + | 0/10 | |

* 5-week-old BALB/c mice observed for 6 months.
† Injected intrathymically.
‡ 4 Gy whole-body.

*Anti-θ serum.*—The serum was produced following Reif and Allen's procedure (1966) of repeated injections of C3H/eB thymocytes into AKR/J mice. Before use, it was absorbed in a liver powder prepared from C3H/eB mice. The resulting antiserum killed all the thymocytes at a 1:64 dilution. For the anti-$\theta$ treatment, cells were incubated in an ice bath for 1 h with the antiserum, then washed and reincubated with guinea-pig complement (lyophilized complement, GIBCO) for 30 min at 37°C. Control cells were incubated with normal AKR/J serum and complement.

*Peanut agglutinin cell fractionation.*—Separation of thymus cells with peanut agglutinin (PNA) was performed according to the method of Reisner *et al.* (1976). Briefly, $10^8$ cells in 0·25 ml of PBS were mixed with 1 mg of PNA in 0·25 ml in a small Falcon plastic tube and left at room temperature for 10 min. The mixture was then gently layered with a Pasteur pipette on the top of a 50% FCS solution in PBS. When the agglutinated fraction settled on the bottom of the tube, it was carefully aspirated with a Pasteur pipette. The upper layer of non-agglutinated thymocytes was removed with a Pasteur pipette into another tube. The lectin was removed from both fractions by a 10min incubation in 2 ml of 0·3M D-galactose. The separated cells were washed twice in PBS, resuspended in MEM and brought to the required cell concentration. The viability of cells was checked by trypan-blue exclusion.

*Winn test.*—The action of lymphoid cells on tumour growth was tested by the Winn assay (Winn, 1961). Lymphoid cells and tumour cells were prepared at the desired concentrations and mixed immediately before injection. The cells were inoculated s.c. into the interscapular space of normal, syngeneic mice. A control group of mice received tumour cells alone. The animals were regularly palpated and the appearance of tumours recorded. Tumours could be detected by palpation when their size was 2–3 mm in diameter.

*Irradiation.*—Irradiation was provided by a Cobalt 60$\gamma$ source (Atomic Energy Ltd, Canada; $\gamma$ beam 15nm, dose rate 10Gy/min). Cells were irradiated in MEM suspension immediately before their use in the Winn test.

*Statistical analysis.*—The statistical significance of the results was analysed by the non-parametric, ranking test of Wilcoxon. Values of $P<0{\cdot}05$ were considered significant.

### RESULTS

*Effect of normal lymphoid cells on the* in vivo *growth of syngeneic lymphoma cells*

The administration of lymphoma cells mixed with cells obtained either from spleen or from thymus of normal mice consistently caused tumour enhancement. As shown in Table II, the latency period of tumour growth was significantly shortened by the addition of lymphoid cells. Tumour enhancement was greater if the lymphocyte:tumour cell ratio was increased (Table II, Exp. 3). If the number of lymphoma cells was reduced to suboptimal doses, the enhancement was manifested by an increase of the final tumour incidence (Table II, Exp. 4, 5 and 8). Tumours were seen in mice injected with as few as $10^2$ lymphoma cells mixed with spleen cells (Table II, Exp. 5). Since $\sim 10^4$ lymphoma cells are required to cause a 100% tumour incidence when

TABLE II.—*Enhancement of lymphoma growth by lymphoid cells of normal mice*

| Exp. | Cells injected† No. of tumour cells | No. of lymphoid cells | Tumour: lymphoid cell ratio | Mean day of tumour appearance ± s.e. (tumour take) | Mean survival time after tumour appearance (days) |
|---|---|---|---|---|---|
| | | | Spleen | | |
| 1 | $10^5$ | — | | 21 ± 1 (9/9) | |
| | $10^5$ | $5\times10^6$ | 1:50 | 16 ± 0 (9/9)***‡ | |
| 2 | $10^5$ | — | | 22 ± 2 (9/9) | 24·6 |
| | $10^5$ | $5\times10^6$ | 1:50 | 16 ± 1 (9/9)* | 24·2 |
| 3 | $10^5$ | — | | 21 ± 1 (10/10) | |
| | $10^5$ | $10^6$ | 1:10 | 17 ± 1 (9/9)* | |
| | $10^5$ | $10^7$ | 1:100 | 15 ± 1 (10/10)*** | |
| 4 | $10^3$ | — | | 21 (1/5) | |
| | $10^3$ | $5\times10^6$ | 1:5000 | 16 ± 0 (5/5)*** | |
| 5 | $10^2$ | — | | 31 (1/10) | |
| | $10^2$ | $5\times10^6$ | 1:50,000 | 24 ± 2 (8/8)*** | |
| | | | Thymus | | |
| 6 | $10^5$ | — | | 24 ± 0 (9/9) | |
| | $10^5$ | $5\times10^6$ | 1:50 | 19 ± 0 (9/9)** | |
| 7 | $10^4$ | — | | 25 ± 3 (5/5) | |
| | $10^4$ | $5\times10^6$ | 1:500 | 16 ± 0 (8/8)** | |
| 8 | $10^3$ | — | | — (0/5) | |
| | $10^3$ | $5\times10^6$ | 1:5000 | 24 ± 2 (5/5)*** | |
| | | | Lymphnode | | |
| 9 | $5\times10^4$ | — | | 24 ± 1 (8/9) | |
| | $5\times10^4$ | $2{\cdot}5\times10^6$ | 1:50 | 23 ± 1 (8/9) n.s. | |

† Cells were injected s.c. into syngeneic normal BALB/c mice. The tumour was a radiation-induced lymphoma of BALB/c mice. Donors of lymphoid cells were normal BALB/c mice.

‡ Wilcoxon test $P$ values calculated *vs* the control group of each experiment (injected with tumour cells alone).

***, $P<0{\cdot}01$; **, $P<0{\cdot}02$; *, $P<0{\cdot}05$; n.s. = not significant.

FIGURE—Transplantability of graded doses of lymphoma cells (s.c. injection into normal syngeneic BALB/c mice).

injected alone (Figure), the addition of $5 \times 10^6$ lymphoid cells increases 100-fold the tumorigenic capacity of lymphoma cells.

Once the tumours were palpable, we could not detect any difference in volume-doubling time between mice injected with tumour cells alone and those injected with tumour cells plus lymphoid cells (data not shown). This is in agreement with the observation that the interval between tumour appearance and death was not modified by the presence of lymphocytes in the inoculum (Table II, Exp. 2) suggesting that the tumour-enhancing effect is restricted to the latent period. In a single experiment (Table II, Exp. 9) peripheral lymphnode cells, in contrast to spleen and thymus cells, caused no significant enhancement of lymphoma growth.

The ability of lymphoid cells to increase the tumorigenic capacity of *in vitro* irradiated lymphoma cells was then examined. As seen in Table III, spleen cells of normal donors accelerated tumour appearance from tumour cells irradiated with a dose of 10 Gy. Moreover, these lymphoid cells allowed the growth of tumour inocula made non-tumorigenic by doses of 40 and 100 Gy. The transfer of an

TABLE III.—*Effect of spleen cells from normal mice on growth of irradiated lymphoma cells**

| Radiation dose to lymphoma cells (Gy) | Final tumour incidence (Mean day of tumour app. ± s.e.) Lymphoma cells only | Lymphoma cells + spleen cells |
|---|---|---|
| — | 5/5 (19 ± 1) | 5/5 (14 ± 1) |
| 10 | 5/5 (32 ± 3) | 5/5 (18 ± 1) |
| 40 | 0/5 | 5/5 (21 ± 1) |
| 100 | 0/5 | 5/5 (24 ± 1) |

* $2 \times 10^5$ lymphoma cells injected s.c. either alone or with spleen cells into syngeneic BALB/c mice. Tumour: spleen cell ratio 1 : 50.

oncogenic virus from the irradiated lymphoma cells to the lymphocytes is certainly not relevant to these results, because of the long latent period of RadLV-induced lymphomas (Haran-Ghera *et al.*, 1977; and see Table I) and because tumour cells derived from a mixture of irradiated lymphoma cells and (BALB/c × C57BL/6)$F_1$ lymphocytes grew in BALB/c hosts and were negative for the H-$2^b$ marker as tested by anti-H-$2^b$ and complement cytotoxicity (data not shown).

In additional experiments, we investigated whether tumour enhancement was detectable in immunosuppressed hosts. For this purpose, mice received whole-body irradiation (6 Gy) and were subsequently challenged with lymphoma cells with or without thymus cells. As seen in Table IV, irradiation produced no significant change in the number of tumour takes and time to tumour appearance in mice injected with tumour alone. More-

TABLE IV.—*Effect of whole-body irradiation on the growth of lymphoma cells*

| Lymphoma cells* | Thymus cells† | Irradiation‡ | Mean day of tumour appearance ± s.e. (final tumour incidence) | |
|---|---|---|---|---|
| $5 \times 10^4$ | — | − | 18 ± 0 (5/5) | |
| $5 \times 10^4$ | — | + | 17 ± 0 (5/5) | |
| $10^4$ | — | − | 24 ± 2 (5/5) | $P < 0.05$ |
| $10^4$ | $5 \times 10^6$ | − | 18 ± 1 (8/8) | |
| $10^4$ | — | + | 25 ± 2 (5/5) | $P < 0.02$ |
| $10^4$ | $5 \times 10^6$ | + | 16 ± 0 (8/8) | |

* Injected s.c. into syngeneic BALB/c mice.

† From normal BALB/c mice.

‡ 6Gy whole-body 24 h before tumour challenge.

over, when lymphoma and thymus cells were injected together, enhancement was equal in normal and irradiated mice. These results suggest that, under these experimental conditions, the growth of the lymphoma is not affected by immunosuppressive procedures such as irradiation.

TABLE V.—*Enhancement of lymphoma growth by thymus cells of different genetic origin*

| Cells injected* | | | |
|---|---|---|---|
| Thymus cells ($5 \times 10^6$) | Origin | Final tumour incidence | Mean day of tumour appearance ± s.e. |
| − | | 0/10 | |
| + | BALB/c | 6/10 | (20 ± 3) |
| + | BALB.B | 4/10 | (19 ± 2) |
| + | C57BL/6 | 6/10 | (19 ± 2) |

* $10^4$ tumour cells derived from a radiation-induced lymphoma of BALB/c mice were injected either alone or together with thymus cells into semi-allogeneic (BALB/c × C57BL/6)-$F_1$ hosts. In these mice, the lymphoma $TD_{50}$ is higher than in BALB/c mice. This explains why no tumour takes were obtained with $10^4$ lymphoma cells alone in this experiment.

As seen in Table V, enhancement was equally evident with syngeneic and allogeneic (H-$2^b$) thymus cells on BALB/c (BALB.B) or C57BL/6 backgrounds, indicating that the genetic origin of thymus cells is not relevant to enhancement. (BALB/c × C57BL/6)$F_1$ mice were used as recipients in this experiment in order to avoid the possibility of host-*vs*-graft reaction against H-$2^b$-bearing lymphocytes.

Whether or not the injection of parental thymus cells causes a GvH reaction, hence host immunosuppression, is unlikely to affect lymphoma growth, given the results previously shown with radiation-immunosuppressed mice.

*Role of T lymphocytes in the enhancement of lymphoma growth*

Previous work on the characterization of tumour-enhancing lymphocytes has emphasized the predominant involvement of T cells in various tumour models (Umiel & Trainin, 1974; Gabizon *et al.*, 1976; Small, 1977). It was important, therefore, to check whether spleen T cells are involved in the enhancement of lymphoma growth in the present experimental model. The results presented in Table VI indicate that treatment with anti-$\theta$ serum totally abolished the enhancing effect of spleen cells from normal mice, and that spleen cells from athymic (*nu*/*nu*) mice were significantly less effective than spleen cells from their heterozygous littermates (*nu*/+) in tumour enhancement. These results, together with the enhancing activity of thymus cells (see Table II, Exp. 6–8) point to a predominant role of thymus-derived cells in the enhancement of lymphoma growth.

TABLE VI.—*Involvement of spleen T cells in enhancement of lymphoma growth**

| Cells injected | Mean day of tumour appearance ± s.e.§ | P |
|---|---|---|
| Tumour only | 24 ± 1 | } 0·02 |
| Tumour + normal serum and C′-treated spleen† | 18 ± 0 | } 0·02 |
| Tumour + anti-$\theta$ and C′-treated spleen† | 24 ± 1 | |
| Tumour only | 29 ± 1 | } 0·01 |
| Tumour + *nu*/+ spleen‡ | 21 ± 1 | } 0·05 |
| Tumour + *nu*/*nu* spleen‡ | 26 ± 2 | |

* $10^5$ lymphoma cells, either with or without spleen cells were injected s.c. into syngeneic BALB/c mice.
† Tumour: spleen cell 1:25.
‡ Tumour: spleen cell 1:100.
§ In every experiment the final tumour incidence was 9/9.

*Maturity and radiation-sensitivity of tumour-enhancing lymphocytes*

An attempt was made to characterize some features of the tumour-enhancing lymphocytes. Thymus cells can be separated into 2 subpopulations by peanut agglutinin (PNA) (Reisner *et al.*, 1976). The cells which bind to PNA are characteristically immature lymphocytes with a low immunological capacity, whereas those which do not bind to PNA are mature and immunocompetent lympho-

TABLE VII.—*Effect of fractionation with PNA on tumour enhancement by thymus cells*

| Cells injected | Mean day of tumour appearance ± s.e.§ | P‡ |
|---|---|---|
| Lymphoma* only | 25 ± 2 | |
| Lymphoma + unfractionated thymus cells† | 16 ± 0 | < 0·01 |
| Lymphoma + $PNA^+$ thymus cells† | 16 ± 1 | < 0·02 |
| Lymphoma + $PNA^-$ thymus cells† | 23 ± 2 | n.s. |

* $10^4$ lymphoma cells were injected s.c. with or without thymus cells into syngeneic BALB/c mice.
† Lymphoma: thymus cell ratio 1 : 500.
‡ Comparison with control (lymphoma only).
§ In every experiment the final tumour incidence was 100%.

cytes apparently equivalent to the cortisone-resistant thymic cells (Reisner *et al.*, 1976). In order to test whether lymphoma enhancement is caused selectively by one of these subpopulations, we fractionated thymus cells with PNA and tested them in the Winn assay. As seen in Table VII, while $PNA^+$ cells were capable of causing enhancement, no significant modification of tumour appearance was found with $PNA^-$ cells. This suggests that tumour enhancement is characteristic of a subpopulation of immature T cells.

Finally, the effect of radiation on the tumour-enhancing capacity of lymphoid cells was investigated. As shown in Table VIII, the tumour-enhancing activity of spleen cells was suppressed by 8 Gy and 20 Gy irradiation when $10^5$ lymphoma cells were injected. It was also found that the increased tumour incidence caused by thymus cells when $10^3$ lymphoma cells were injected was gradually cancelled by 8 Gy and 20 Gy irradiation of thymus cells.

### DISCUSSION

The present investigations indicate that T cells from normal mice enhance the growth of lymphoma cells in *in vivo* transfer tests. This effect was consistently found with spleen and thymus cells and was expressed as an earlier tumour appearance, when a supraoptimal number of lymphoma cells was injected, and by a higher tumour incidence, when a suboptimal number of lymphoma cells was injected. Differences of several days in tumour appearance were reflected by striking changes in tumour size, due to the short volume-doubling time of this tumour (2 days). This stresses the biological relevance of the data with supraoptimal doses of lymphoma cells. The enhancing activity of lymphoid cells was also manifested on the growth of irradiated lymphoma cells. The development of tumours from mixtures of irradiated lymphoma cells and lymphoid cells might be explained by an enhancing effect of the latter on a small remaining number of viable tumour cells. This is supported by the fact that the

TABLE VIII.—*Radiosensitivity of the enhancing effect of lymphoid cells on lymphoma growth*

| Cells injected per mouse* | Radiation dose to lymphoid cells (Gy) | Mean day of tumour appearance ± s.e. | Final tumour incidence | P |
|---|---|---|---|---|
| L ($10^5$) | — | 16 ± 0 | (7/7) | } < 0·01 |
| L ($10^5$) + S ($5 \times 10^6$) | — | 12 ± 0 | (7/7) | } < 0·01 |
| L ($10^5$) + S ($5 \times 10^6$) | 8 | 18 ± 0 | (7/7) | } n.s. |
| L ($10^5$) + S ($5 \times 10^6$) | 20 | 17 ± 1 | (7/7) | |
| L ($10^3$) | — | 20 ± 0 | (1/6) | } < 0·01 |
| L ($10^3$) + T ($5 \times 10^6$) | — | 17 ± 1 | (8/8) | } < 0·01 |
| L ($10^3$) + T ($5 \times 10^6$) | 8 | 21 ± 2 | (4/8) | } n.s. |
| L ($10^3$) + T ($5 \times 10^6$) | 20 | — | (0/6) | |

* Lymphoma cells (L) injected s.c. with or without lymphoid cells from spleen (S) or thymus (T) into syngeneic BALB/c mice.

injection of as few as $10^2$ lymphoma cells led to 100% tumour incidence when lymphoid cells were added in the inoculum (Table II). The possibility of a helper effect on the repair capacity of sublethally damaged tumour cells must also be considered, especially since cell-to-cell contacts, which are known to influence the repair process of sublethal injury (Durand & Sutherland, 1972) may occur between lymphoma and lymphoid cells.

Our experiments with anti-$\theta$ serum, and with spleen cells from nude mice, indicate a predominant role of T cells in the enhancement of lymphoma growth. The fractionation of thymocytes with peanut agglutinin further indicates that a subpopulation of immature T cells, rather than the whole thymus-cell population, is responsible for the tumour-enhancement effect, suggesting that the tumour-enhancing properties of T cells are cancelled out during maturation. These results are similar to those of Umiel *et al.* (1978) with other tumours.

The link between immature T lymphocytes and tumour-enhancement is further supported by the experiments of Small (1979) in which TdT activity, a marker of early thymocytes (Barton *et al.*, 1976) was increased in spleen cells of tumour-bearing mice with tumour-enhancing activity. The finding that peripheral lymphnode cells from normal mice showed no tumour-enhancing activity is in agreement with the high level of maturation and immunocompetence of lymphnode T cells (Trainin *et al.*, 1979).

We have shown previously that the enhancing effect of spleen cells from mice bearing a chemically induced fibrosarcoma was not tumour specific (Gabizon *et al.*, 1976). Together with this, the present observations indicate that a non-sensitized lymphoid population is capable of tumour enhancement, suggesting that a process of specific immunological recognition is not necessarily involved in tumour enhancement. Yet tumour antigens could interact with potentially tumour-enhancing lymphoid cells *in situ* after inoculation of the lymphoid-tumour-cell mixture. However, the lack of difference in the enhancing effects of lymphoid cells of different genetic origin (Table V) argues against the relevance of immunological recognition phenomena. Tumour enhancement by allogeneic, as well as syngeneic, lymphocytes has been shown in other experimental models, including spleen cells of tumour-bearing hosts (Manor *et al.*, 1976) and *in vitro* tumour-sensitized lymphocytes (Small & Trainin, 1975).

The enhancing effect of lymphoid cells was abrogated by irradiation, indicating that tumour enhancement is not due to a simple feeder effect of dead or irradiated cells similar to the Revesz effect (Revesz, 1958) but to an active process of living cells. In addition, we have found no enhancement of lymphoma growth with supernatants of lymphoid cells (data not shown). It is not clear whether tumour enhancement involves suppression of host resistance, or a direct stimulation of tumour cells by transferred lymphocytes. Hellström & Hellström (1978) have presented evidence for the existence of a suppressor, radiosensitive spleen T cell in normal and tumour-bearing mice, which enhances tumour growth. However, the possibility of an immunostimulatory effect was not totally discarded (Hellström *et al.*, 1978). As shown in this paper, the fact that lymphoid cells could still enhance tumour growth in radiation-immunosuppressed animals is against a suppressive mechanism. A similar result was reported by Carnaud *et al.* (1974) with another tumour model, suggesting tumour stimulation by enhancing lymphoid cells. The nature of a putative stimulatory interaction between lymphoma and T cells has not been investigated, but a specific response of this T-cell-derived tumour to growth factors of T-cell origin (Morgan *et al.*, 1976) should be considered.

The phenomenon of tumour enhancement may be especially relevant to lymphomas rather than other solid, non-lymphoma tumours. Most mouse lymphomas originate and metastasize in the vicinity of large concentrations of lymphoid cells, including T cells. There is a possibility that small nests of transformed cells are stimulated to proliferate and grow into overt clinical disease in environments such as the thymus by a similar interaction to that found in the present experiments.

We thank Professor N. Haran-Ghera for helpful discussions during the course of this work.

Nathan Trainin is the Harold L. Korda Professor of Cancer Research.

### REFERENCES

BARTON, R., GOLDSCHNEIDER, I. & BOLLUM, F. J. (1976) The distribution of terminal deoxynucleotidyl transferase (TdT) among subsets of thymocytes in the rat. *J. Immunol.*, **116**, 462.

CARNAUD, C., ILFELD, D., LEVO, Y. & TRAININ, N. (1974) Enhancement of 3LL tumor growth by autosensitized T lymphocytes independent of the host lymphatic system. *Int. J. Cancer*, **14**, 168.

DECKERS, P. J., RAMMING, K. P. & PILCH, Y. H. (1971) Facilitation of tumor growth by syngeneic normal cells mixed with tumor cells in vitro. *Cancer*, **27**, 897.

DURAND, R. E. & SUTHERLAND, R. M. (1972) Effects of intercellular contact on repair of radiation damage. *Exp. Cell Res.*, **71**, 75.

FIDLER, I. J. (1973) *In vitro* studies of cellular mediated immunostimulation of tumor growth. *J. Natl Cancer Inst.*, **50**, 1307.

FIDLER, I. J. (1974) Immune stimulation–inhibition of experimental cancer metastases. *Cancer Res.*, **34**, 491.

GABIZON, A., SMALL, M. & TRAININ, N. (1976) Kinetics of the response of spleen cells from tumor-bearing animals in an *in vivo* tumor-neutralization assay. *Int. J. Cancer*, **18**, 813.

GILLETTE, R. W., BERRINGER, D. C. & WUNDERLICH, D. A. (1978) Resistance to syngeneic lymphoma cells as a result of immunization with chemically-modified allogeneic lymphoma cells in mice. *J. Natl Cancer Inst.*, **60**, 1427.

GILLETTE, R. W. & FOX, A. (1975) The effect of T lymphocyte deficiency on tumor induction and growth. *Cell. Immunol.*, **19**, 328.

HARAN-GHERA, N., BEN-YAAKOV, M. & PELED, A. (1977) Immunologic characteristics in relation to high and low leukemogenic activity of radiation leukemia virus variants. I. Cellular analysis of immunosuppression. *J. Immunol.*, **118**, 600.

HELLSTRÖM, K. E. & HELLSTRÖM, I. (1978) Evidence that tumor antigens enhance tumor growth *in vivo* by interacting with a radiosensitive (suppressor) cell population. *Proc. Natl Acad. Sci., U.S.A.*, **75**, 436.

HELLSTRÖM, K. E., HELLSTRÖM, I., KANT, J. A. & TAMERIUS, J. D. (1978) Regression and inhibition of sarcoma growth by interference with a radiosensitive T-cell population. *J. Exp. Med.*, **148**, 799.

ILFELD, D., CARNAUD, C., COHEN, I. R. & TRAININ, N. (1973) *In vitro* cytotoxicity and *in vivo* tumor enhancement induced by mouse spleen cells autosensitized *in vitro*. *Int. J. Cancer*, **12**, 213.

MANOR, Y., TREVES, A. J., COHEN, I. R. & FELDMAN, M. (1976) Transition from T-cell protection to T-cell enhancement during tumor growth in an allogeneic host. *Transplantation*, **22**, 360.

MORGAN, D. A., RUSCETTY, F. W. & GALLO, R. (1976) Selective *in vitro* growth of T lymphocytes from normal human bone marrows. *Science*, **193**, 1007.

PREHN, R. T. (1972) The immune reaction as a stimulator of tumor growth. *Science*, **176**, 170

PREHN, R. T. (1976) Do tumors grow because of the immune response of the host? *Transplant. Rev.*, **28**, 34.

REIF, A. E. & ALLEN, J. M. (1966) Mouse thymic isoantigens. *Nature*, **209**, 521.

REISNER, Y., LINKER-ISRAELI, M. & SHARON, N. (1976) Separation of mouse thymocytes into two subpopulations by the use of peanut agglutinin. *Cell. Immunol.*, **25**, 129.

REVESZ, L. (1958) Effect of lethally damaged tumor cells upon the development of admixed viable cells. *J. Natl Cancer Inst.*, **20**, 1157.

RYGAARD, J. & POVLSEN, C. O. (1976) The nude mouse *vs* the hypothesis of immunological surveillance. *Transplant. Rev.*, **28**, 43.

SMALL, M. & TRAININ, N. (1975) Inhibition of syngeneic fibrosarcoma growth by lymphocytes sensitized on tumor cell monolayers in the presence of the thymic humoral factor. *Int. J. Cancer*, **15**, 962.

SMALL, M. & TRAININ, N. (1976) Separation of populations of sensitized lymphoid cells into fractions inhibiting and fractions enhancing syngeneic tumor growth *in vivo*. *J. Immunol.*, **117**, 292.

SMALL, M. (1977) Characteristics of the immature cells involved in T-cell-mediated enhancement of syngeneic tumor growth. *J. Immunol.*, **118**, 1517.

SMALL, M. (1979) Release of immature cells from the thymus during solid tumor growth: Identification by assay of TdT activity. *J. Immunol.*, **123**, 259.

TRAININ, N., YAKIR, Y. & KOOK, A. I. (1979) Correlation between the cellular levels of cAMP, cell-mediated immunocompetence and the thymic humoral factor (THF). In: *Cell Biology and Immunology of Leukocyte Function*. Acad. Press, Inc. p. 201.

UMIEL, T. & TRAININ, N. (1974) Immunological enhancement of tumor growth by syngeneic thymus-derived lymphocytes. *Transplantation*, **18**, 244.

UMIEL, T., LINKER-ISRAELI, M., ITSCHAKI, M., TRAININ, N., REISNER, Y. & SHARON, N. (1978) Inhibition or acceleration of tumor growth by subpopulations of thymus cells separable by a peanut lectin. *Cell. Immunol.*, **37**, 134.

WINN, H. J. (1961) Immune mechanisms in homotransplantation. II. Quantitative assay of the immunologic activity of lymphoid cells stimulated by tumor homografts. *J. Immunol.*, **86**, 228.